# Selective enhancement of cardiomyocyte efficiency results in a pernicious heart condition

**DOI:** 10.1371/journal.pone.0236457

**Published:** 2020-08-13

**Authors:** Jody Groenendyk, Qian Wang, Cory Wagg, Dukgyu Lee, Alison Robinson, Amy Barr, Peter E. Light, Gary D. Lopaschuk, Luis B. Agellon, Marek Michalak

**Affiliations:** 1 Department of Biochemistry, University of Alberta, Edmonton, Alberta, Canada; 2 Department of Pharmacology, University of Alberta, Edmonton, Alberta, Canada; 3 Department of Pediatrics, University of Alberta, Edmonton, Alberta, Canada; 4 School of Human Nutrition, McGill University, Ste. Anne de Bellevue, Quebec, Canada; University of Cincinnati College of Medicine, UNITED STATES

## Abstract

Transgenic mice with selective induction of calreticulin transgene expression in cardiomyocytes (Cardiac^CRT+^) were analyzed. Cardiac^CRT+^ cardiomyocytes showed increased contractility and Ca^2+^ transients. Yet, *in vivo* assessment of cardiac performance, and ischemic tolerance of Cardiac^CRT+^ mice demonstrated right ventricle dilation and reduced cardiac output, increased QT interval and decreased P amplitude. Paradoxically, *ex vivo* working hearts from Cardiac^CRT+^ mice showed enhanced ischemic cardio-protection and cardiac efficiency. Under aerobic conditions, Cardiac^CRT+^ hearts showed less efficient cardiac function than sham control hearts due to an increased ATP production from glycolysis relative to glucose oxidation. During reperfusion, this inefficiency was reversed, with Cardiac^CRT+^ hearts exhibiting better functional recovery and increased cardiac efficiency compared to sham control hearts. On the other hand, mechanical stretching of isolated cardiac fibroblasts activated the IRE1α branch of the unfolded protein response pathway as well as induction of Col1A2 and TGFβ gene expression *ex vivo*, which were all suppressed by tauroursodeoxycholic acid.

## Introduction

The heart is a multicellular organ that takes advantage of the functions of specialized cell types (cardiomyocytes, fibroblasts, macrophages, Purkinje and pacemaker, endothelial) to carry on mechanical work, tissue remodeling and energetics [1]. For example, cardiomyocytes are primarily responsible for the cardiac mechanical function and cardiac fibroblasts are involved in tissue remodeling [1–4]. Heart failure typically results from abnormally long periods of extreme mechanical activity [5] and cardiac remodeling induces fibrogenesis, initially an adaptive response, which leads to cardiac pathology. Furthermore, the heart is an energy-demanding organ and many cardiac diseases such as cardiac hypertrophy, heart failure, myocardial ischemia are associated with fluctuations in cardiac energy metabolism [6, 7]. In particular, a decrease in mitochondrial oxidative metabolism and glucose oxidation occurs, with a greater reliance of the heart on glycolysis as a source of energy [7]. An increased reliance on fatty acid oxidation compared to glucose oxidation, and an uncoupling of glycolysis from glucose oxidation results in a decrease in cardiac efficiency in the hypertrophied and failing heart.

Ca^2+^ is integral to cardiac development, physiology and pathology, and Ca^2+^ handling proteins associated with the sarcoplasmic reticulum (SR) and endoplasmic reticulum (ER) membranes in cardiomyocytes are critical for excitation-contraction coupling in the heart, as well as for housekeeping functions in the cell [8]. Calreticulin, a major ER Ca^2+^ binding protein, is highly expressed in the embryonic heart and is necessary for normal cardiac development [9]. After birth, the calreticulin gene is sharply downregulated in the heart, and thus, adult hearts have very low levels of this protein [9, 10]. Previous studies noted increased expression of calreticulin in cardiac hypertrophy [11–14] and in the failing human heart [15], and we showed experimentally that mice with upregulated expression of calreticulin in adult cardiomyocytes develop dilated cardiomyopathy, cardiac remodeling (fibrogenesis) and heart failure [16, 17]. Induction of expression of calreticulin is associated with increased Ca^2+^ concentration in the ER lumen, doubled rate of ER Ca^2+^ refilling and enlarged release of Ca^2+^ from stimulated cells [18]. Furthermore, adult hearts with increased expression of calreticulin have altered expression of Ca^2+^ handling proteins (RyR, SERCA, triadin and calsequestrin), connexin43 and connexin45 [16, 17], illustrating a role for calreticulin in modulating cardiomyocyte Ca^2+^ homeostasis. Altered Ca^2+^ homeostasis in cardiomyocytes with increased abundance of calreticulin likely contributes to calreticulin-induced cardiac pathology. Interestingly, cardiac fibrosis in adult mice overexpressing calreticulin in cardiomyocytes can be prevented by administration of TUDCA, a proteostasis promoter [19] which inhibits the IRE1α signaling arm of the unfolded protein response (UPR) pathway, an ER stress coping response [17]. Whether these changes are caused by failure of myocardial contraction or other adaptive responses is not well established.

## Experimental procedures

### Ethics

All animal experiments were carried out in accordance with the Canadian Council on Animal Care Guidelines. The approval for use of animals in research was granted by the Animal Care and Use Committee for Health Sciences, a University of Alberta ethics review committee (Permit AUP297). Animals were monitored daily for responsiveness, body conditions, respiration, physical appearance and mobility. Mice are anesthetized using inhalant anesthetic (Isoflurane). Animals were euthanized by cervical dislocation according to our approved animal protocol when they met specific criteria or showed signs of distress. No animal died prior to experimental endpoints. Total of 260 animals were used in the study (equal number of male and female mice).

### Transgenic mice with increased expression of calreticulin in cardiomyocytes

Generation of cardiac-specific calreticulin transgenic mouse and induction of calreticulin transgene expression in the adult stage (Cardiac^CRT+^) were described previously [16, 17]. Briefly, the CAT-loxP-CRT mice (C57BL/6 background) was cross-bred with αMHC-Cre mice (C57BL/6 background; control) to generate double transgenic mice (αMHC/ CAT-loxP-CRT) [16]. Only male mice were used in all the experiments. To induce calreticulin transgene expression, 80 mg of Tamoxifen was mixed with 200 g of powdered rodent feed and 100 ml of water then formed into small cakes (8–10 g) and fed to mice (control and double transgenic mice) for 3 weeks. Tamoxifen-fed double transgenic mice are referred to throughout the paper as Cardiac^CRT+^ mice while tamoxifen-fed C57BL/6 mice are referred to as sham controls. For the TUDCA experiments, 2 mg/ml TUDCA was added to the drinking water ad libitum during the Tamoxifen treatment [17].

### Measurement of *ex vivo* cardiac function

Heart rates and pressure measurements in isolated working hearts were recorded using a pressure transducer in the aortic outflow line (Harvard Apparatus). Data were collected using an MP100 system from AcqKnowledge (BIOPAC Systems, Inc.). Cardiac output and aortic flows were obtained by measuring the flow into the left atria and from the afterload line, respectively, using Transonic flow probes. Cardiac function was calculated as the product of heart rate x peak systolic pressure. Cardiac power was calculated as the product of cardiac output x LV developed pressure (systolic pressure—preload pressure) x a conversion factor of 1.33 x 10^−4^ as described [20]. Frozen hearts were powdered and ~20 mg (wet weight) samples were dried at 60°C overnight (dry weight). The ratio of this sample (dry/wet weight) was used to calculate the total dry mass of the heart.

### *Ex vivo* working mouse hearts

Hearts from Cardiac^CRT+^ mice and sham control mice were excised and cannulated via the aorta and left atrium immediately after euthanasia. *Ex vivo* perfused working mouse were aerobically perfused with Krebs-Henseleit solution containing 5 mM glucose, 100 μU/ml insulin, 2.5 mM free Ca^2+^, and 1.2 mM palmitate bound to 3% BSA for 30 min followed by 20 min of global no-flow ischemia and 40 min of aerobic reperfusion as described previously [21]. Hearts were perfused with a buffer containing either [5-^3^H/U-^14^C]glucose and unlabeled palmitate (for glycolysis and glucose oxidation measurements), or [U-^14^C]glucose and [9,10-^3^H]palmitate (for glucose and palmitate oxidation measurements). Rates of glucose oxidation, fatty acid oxidation and glycolysis were measured by quantitative collection of either ^3^H_2_O or ^14^CO_2_, as described [21, 22].

### Measurement of energy metabolic rates and calculation of ATP production rates

Rates of glycolysis, glucose oxidation, and fatty acid oxidation were measured and expressed per gram viable tissue (μmol/min/g dry wt) as described previously [23], using [5-^3^H]glucose, [U-^14^C]glucose, [9,10-^3^H]palmitate, and [U-^14^C]lactate, respectively and measuring ^3^H_2_O and ^14^CO_2_ production. ATP production rates from each substrate (25 for palmitate oxidation, 25 for glucose oxidation, and 6 for glycolysis) were calculated.

### ECHO, ECG and ECHO-MRI and histological analyses

ECHO, ECG and ECHO-MRI analysis were carried out as previously described [16, 17, 24]. Briefly, animals were fed tamoxifen for 3 weeks ± 2 mg/ml TUDCA in the drinking water. ECHO and ECG were carried out at Week 1, 2 and 3 followed by sacrifice for *ex vivo* heart function. ECHO-MRI was carried out at Week 3. Staining was performed as previously described [16, 17].

### Metabolic cages

*In vivo* calorimetry was carried out as previously described [25]. Briefly, mice were fed for 3 weeks of Tamoxifen supplemented rodent food ± 2 mg/ml TUDCA in the drinking water, followed by 48 h in CLAMS (lab animal monitoring system) metabolic cages (Columbus Instruments) to monitor VO_2_ and VCO_2_, heat, ambulation and other parameters. CLAX software was used to monitor the animal parameters.

### Isolation of cardiomyocytes

Isolation of cardiomyocytes was performed as previously described using the Langendorff heart perfusion system [26]. Briefly, *ex vivo* hearts were hung on a cannula and perfusion buffer (130 mM NaCl, 5 mM KCl, 0.5 mM NaH_2_PO_4_, 10 mM HEPES, 10 mM glucose, 10 mM BDM, 10 mM Taurine, 1 mM MgCl_2_, pH 7.8), followed by collagenase digestion buffer (perfusion buffer with 0.5 mg/ml Collagenase 2, 0.5 mg/ml Collagenase 4, and 0.05 mg/ml Protease XIV) was perfused throughout the heart. Cardiomyocytes were isolated from the perfused heart by gently teasing the tissue apart, filtering the cell suspension through a 100 μm strainer and allowing the cardiomyocytes to sediment by gravity. Ca^2+^ was reintroduced (by gradual increments) to the cardiomyocytes, followed by plating onto laminin coated plates in culture media [M199 medium, 0.1% BSA, 1X ITS (insulin, transferrin, selenium), 10 mM BDM, 1X CD lipid (chemically defined lipid concentrate), and 10 U/ml Penicillin, and 100 μg/ml Streptomycin] and incubation at 37°C in 5% CO_2_ incubator [26].

### Ca^2+^ transient and contraction measurements

Isolated cardiomyocytes were plated on 25 mm coverslips in a 60 mm dish, 1 day before measurements. Coverslips were placed in a magnetic holder and cells were washed with ADS buffer (120 mM NaCl, 6 mM glucose, 8 mM NaH_2_PO_4_, 5 mM KCl, 0.8 mM MgSO_4_, 20 mM HEPES, pH 7.4) followed by the addition of 5 μl of 1 mM Calcium Green-1/AM in 1 ml ADS buffer. Cells were incubated for 15 min at room temperature and washed with ADS buffer 2 times. Cells were visualized using an Axiovert S100 microscope with excitation of 506 nm and emission of 531 nm. Eclipse software was used to monitor Ca^2+^ transients. Contraction measurements were performed using an Axiovert S100 microscope with phase contrast, followed by video recording of contractions.

### Isolation and culture of cardiac fibroblasts from adult mice

Adult cardiac fibroblasts were isolated from 6–8 weeks old C57BL/6J male mice. The hearts were perfused using a Langendorff-Free method [27]. Hearts were removed followed by injection into the apex of left ventricle with 7 ml of EDTA buffer containing 130 mM NaCl, 5 mM KCl, 0.5 mM NaH_2_PO_4_, 10 mM Glucose, 10 mM 2,3-butanedione monoxime (BDM), 10 mM Taurine, 5 mM EDTA and 10 mM HEPES, pH 7.8. Then the ascending aorta was clamped using surgical hemostats, and the heart was transferred to a fresh EDTA buffer This was followed by injection, at 2 ml/min, of 10 ml EDTA buffer, then 3 ml perfusion buffer containing 130 mM NaCl, 5 mM KCl, 0.5 mM NaH_2_PO_4_, 10 mM Glucose, 10 mM BDM, 10 mM Taurine, 1 mM MgCl_2_ and 10 mM HEPES, pH 7.8, and 50 ml collagenase buffer containing 0.5 mg/ml Collagenase 2 (Sigma-Aldrich C6885); 0.1 mg/ml Collagenase 4 (Sigma-Aldrich C5138); Protease type XIV, 0.05 mg/ml (Sigma-Aldrich P5147) all dissolved in a perfusion buffer. Cells were dissociated with gentle trituration, and digestion was stopped by addition of 5 ml of perfusion buffer containing 5% FBS. Cells were passed through a 100 μm cell strainer, centrifuged, washed in DMEM (Gibco, 11995) containing 10% FBS and resuspended in 10% FBS DMEM, 10 U/ml Penicillin, and 100 μg/ml Streptomycin and plated onto 10 cm culture dishes (Corning, 430167).

### Extrinsic mechanical stretch on cultured cardiac fibroblasts

Cardiac fibroblasts (5 x 10^5^ cells/well) were cultured on non-coated BioFlex culture plates (Flexcell International Corporation, BF-30001U) in DMEM (Gibco, 11995) supplemented with 10% FBS, 10 U/ml Penicillin, and 100 μg/ml Streptomycin. Experiments were carried out with a second passage of cardiac fibroblasts. The media was changed to serum free DMEM with 10 U/ml Penicillin, and 100 μg/ml Streptomycin 24 h before stretching. The cells were subjected to heart (P) wave cyclic stretch under 1 Hz frequency on the Flexcell strain apparatus (FX-5000, Flexcell International Corporation), with 0% minimum and 10% maximum elongation. The cyclic stretch was performed for 20 h in serum-free DMEM media (control) or serum-free DMEM with 200 μM TUDCA.

### RNA isolation and qPCR

mRNA was isolated from fibroblasts using the RNeasy kit (Qiagen) according to the manufacturer’s protocol. Total RNA (200 ng) was used to synthesize cDNA for use in quantitative PCR (qPCR) [24]. The following oligonucleotide primers were used for qPCR analyses:

18s: Forward 5’-AACCCGTTGAACCCCATT-3’, Reverse 5’-CCATCCAATCGGTAGTAGCG-3’

sXBP1 (spliced XBP1); Forward 5’-GAGTCCGCAGCAGGTG-3’, Reverse 5’-GTGTCAGAGTCCATGGGA-3’

Col1A2 (collagen type I, α2): Forward 5’- CCAGCGAAGAACTCATACAGC -3’, Reverse 5’- GGACACCCCTTCTACGTTGT -3’

TGFβ1 (transforming growth factor β1: Forward 5’-CACCTGCAAGACCATCGACAT-3’, Reverse 5’-GAGCCTTAGTTTGGACAGGATCTG-3’

### Statistical analysis

All data are presented as mean ± SEM. Comparison between two groups was performed using a Paired Student t-test. Differences were considered significant when p value<0.05. Statistical analysis was performed using GraphPad Prism 8.

## Results

### Mice with targeted and forced overexpression of calreticulin in cardiomyocytes

We previously showed that increased expression of calreticulin in cardiomyocytes of adult mouse hearts (Cardiac^CRT+^ hearts) consistently induces dilated cardiomyopathy, extensive cardiac fibrosis and heart failure (S1A Fig, S1B Fig) [16, 17]. Induction of calreticulin overexpression is accompanied by transient activation of the IRE1α signaling arm of the UPR pathway before the appearance of pathology [17]. Inhibition of the IRE1α -dependent Xbp1 mRNA splicing by tauroursodeoxycholic acid (TUDCA) in the Cardiac^CRT+^ mice remarkably prevents cardiac fibrogenesis [17]. On the other hand, TUDCA treatment had no effect on cardiac function of the failing heart (S1A Fig, S1B Fig) [17]. Cardiac^CRT+^ mice whether treated or not with TUDCA also had reduced cardiac output and ejection fraction, as well as increased myocardial performance index (Tei index) (S1B Fig; Table 1), irregular echocardiography including decreased QRS amplitude and T amplitude [16] (S1C Fig; Table 2).

**Table 1:** Echocardiography of sham control and Cardiac^CRT+^ mice.

Measurement	Sham control	Cardiac^CRT+^	Cardiac^CRT+^+TUDCA
Body weight (g)	22.3±1.0	20.7±0.6	19.7±0.0
Heart rate (bpm)	426.4±26.8	460.9±26.9	461.0±8.1
IVSd (mm)	0.7±0.0	0.6±0.0	0.6±0.0
LVIDd (mm)	4.1±0.1	4.6±0.1	4.9±0.0
LVPWd (mm)	0.7±0.0	0.6±0.0	0.6±0.0
Cardiac output (ml/min)	16.1±1.4	10.5±1.2	5.0±0.4
%EF	56.4±4.8	22.8±4.4	10.2±0.4
Mitral E velocity (mm/sec)	641.2±34.8	482.2±99.9	400.8±67.3
Mitral E/A ratio	1.7±0.2	4.0±0.8	N/A
TEI index	0.7±0.2	1.0±0.1	1.1±0.0
d wave (mm/sec)	478.7±28.0	180.9±38.9	101.8±1.0
s/d ratio	0.4±0.0	0.2±0.0	0.1±0.0
s’	17.8±1.5	10. ±1.1	7.4±0.9

Values are mean ± SEM, LV, left ventricle; IVSd, intraventricular septum diastolic; LVIDd, left ventricle inner diameter diastolic; LVPWd, left ventricle posterior wall diastolic; %EF, percentage of ejection fraction; Tei index, an index of myocardial performance in systolic and diastolic function.

**Table 2:** ECG analysis of sham control and Cardiac^CRT+^ mice.

Measurement	Sham control	Cardiac^CRT+^	Cardiac^CRT+^+TUDCA
Body weight (g)	24.7±1.1	21.6±1.16	20.1±0.8
Heart rate (bpm)	386±028	485±94	395±117
PR interval (sec)	0.16±0.01	0.14±0.02	0.16±0.05
P duration (sec)	0.035±0.005	0.031±0.005	0.036±0.002
QRS interval (sec)	0.011±0.001	0.012±0.002	0.012±0.001
QT interval	0.021±0.004	0.029±0.005	0.029±0.007
QTc (sec)	0.054±0.013	0.081±0.01	0.077±0.029
JT interval (sec)	0.01±0.005	0.016±0.005	0.017±0.009
T peak-T end interval (sec)	0.007±0.017	0.011±0.004	0.014±0.008
P amplitude (mV)	0.049±0.2	0.01±0.006	0.014±0.008
Q amplitude (mV)	-0.17±0.066	-0.493±0.033	-0.053±0.003
R amplitude (mV)	0.914±0.279	1.011±30.494	0.453±0.005
S amplitude (mV)	-0.250±0.082	-0.526±0.37	-0.157±0.056

Values are mean ± SEM

### Reduced rate of physical activity and increased respiratory exchange ratio in Cardiac^CRT+^ mice

**Fig 1:**
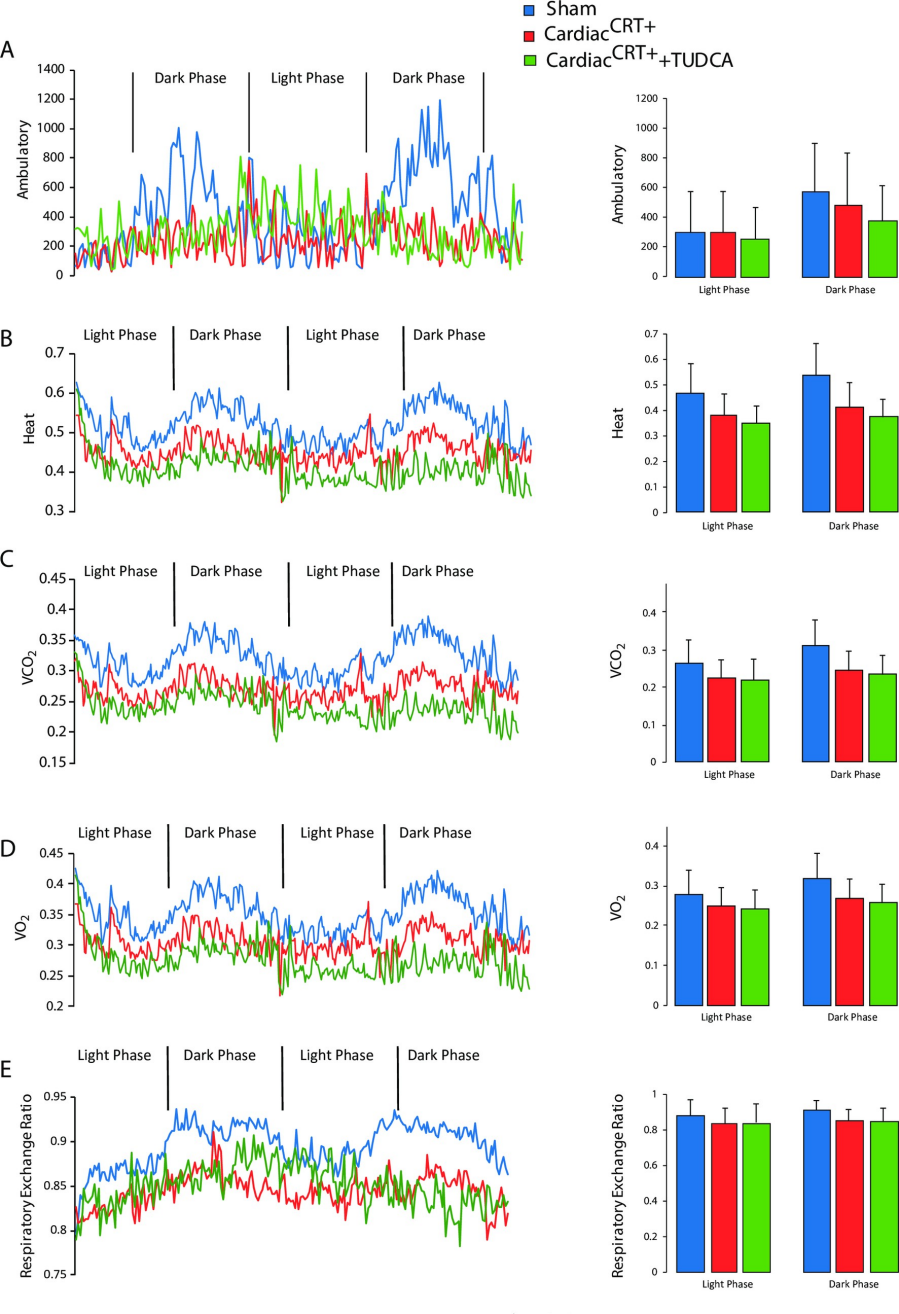
Calorimetric analysis of transgenic animals. **A.** Ambulatory rates over 48 h. **B.** Heat was calculated from calorific value (CV) that shows the relationship between heat and the volume of oxygen consumed. *CV* = 3.815 + 1.232 * RER. Heat = *CV* * VO_2_. **C.** Volume carbon dioxide production (VCO_2_). **D.** Volume oxygen consumption (VO_2_) value. **E.** Respiratory exchange ratio (RER) over 48 h. RER was calculated from VCO_2_/VO_2_. An RER of 1.00 indicates utilization of pure carbohydrate as an energy source and an RER of 0.70 indicates utilization of pure fat as an energy source. Average ambulatory rates, heat, VCO_2_, VO_2_ and Respiratory Exchange Rates (RER) are shown on the right. Sham control (n = 6), Cardiac^CRT+^ (n = 6), Cardiac^CRT+^+TUDCA (n = 6), values are mean ± SEM. *p value<0.0001.

To gain further insight into the nature of the cardiac defect in Cardiac^CRT+^ mice, we first carried out analysis of metabolic rate by indirect calorimetry. Consistent with our earlier finding [17], administration of TUDCA resulted in a 30% increase in ambulation rate of Cardiac^CRT+^ mice especially during the light phase (Fig 1A). Moreover, TUDCA treatment maintained body temperature, VCO_2_, and VO_2_, which were reduced in untreated Cardiac^CRT+^ mice especially during the dark phase (Fig 1B–1D). Cardiac^CRT+^ mice were less active as compared to sham controls during the dark phase (Fig 1A). We measured the respiratory exchange ratio (RER) of Cardiac^CRT+^ mice to assess the preferred energy substrate and revealed that during the dark phase Cardiac^CRT+^ mice preferably utilized carbohydrate as an energy source whereas sham controls preferred fat (Fig 1E).

### Efficiency of cardiac energy metabolism in Cardiac^CRT+^ hearts

**Fig 2:**
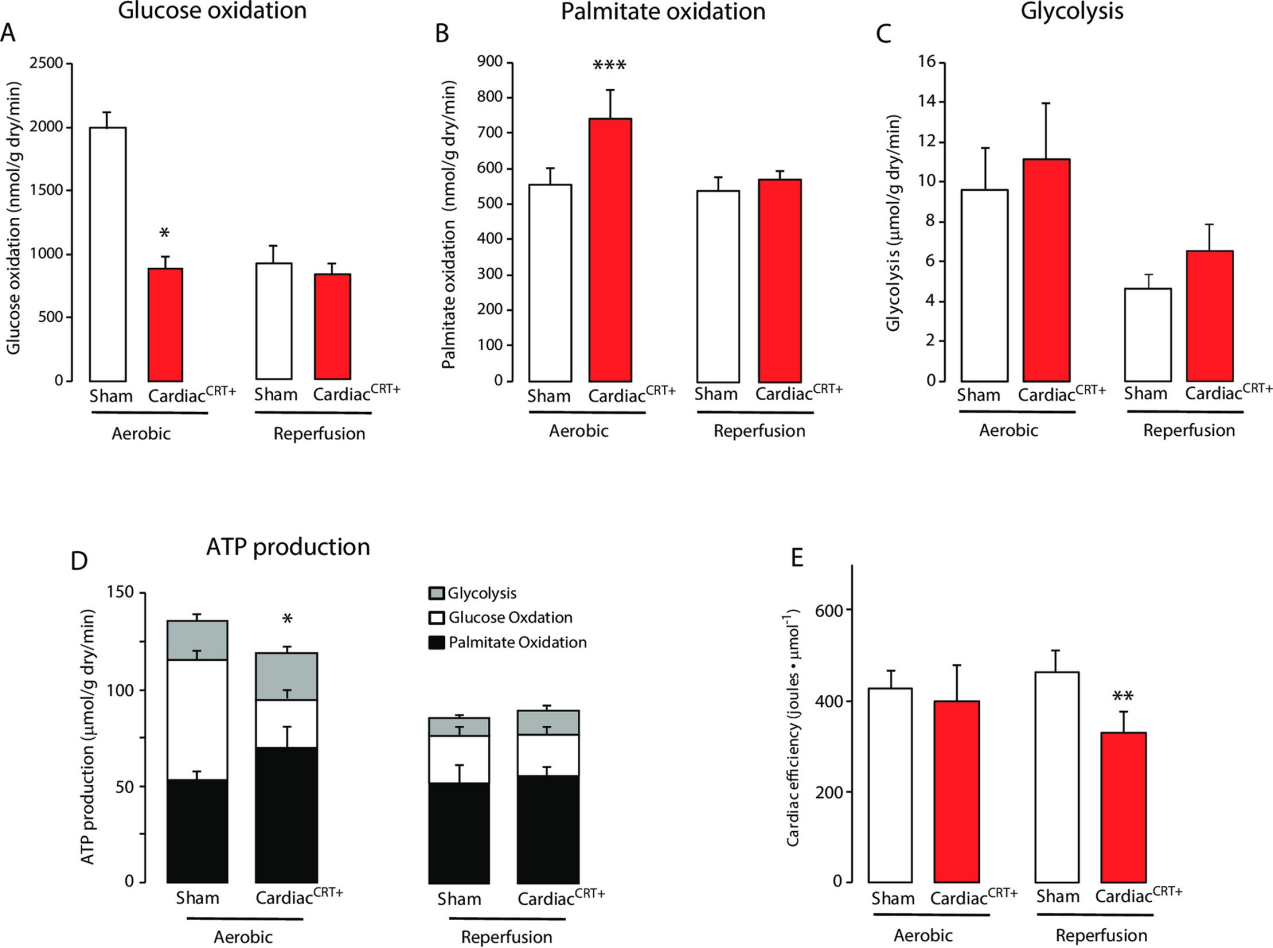
Metabolic rates and ATP production in aerobic perfusion. Cardiac^CRT+^ and sham control hearts were subjected to work *ex vivo* to measure rates of glucose oxidation (**A**), palmitate oxidation (**B**), and glycolysis (**C**). **D.** Measurement of proportional yield of ATP production in glucose oxidation, palmitate oxidation and glycolysis during aerobic perfusion. Values within the bars represent ATP production in μmol ATP/g dry wt^-1^/min^-1^ (n = 6). **E.** Ratio of the cardiac work/ATP production during aerobic perfusion. Values represent metabolic rates measured during an initial aerobic perfusion (Aerobic), or during the aerobic reperfusion following global ischemia (Reperfusion). *p value<0.0001, **p value = 0.0027, ***p value = 0.062, n = 20 for sham controls and n = 25 for Cardiac^CRT+^ mice.

**Fig 3:**
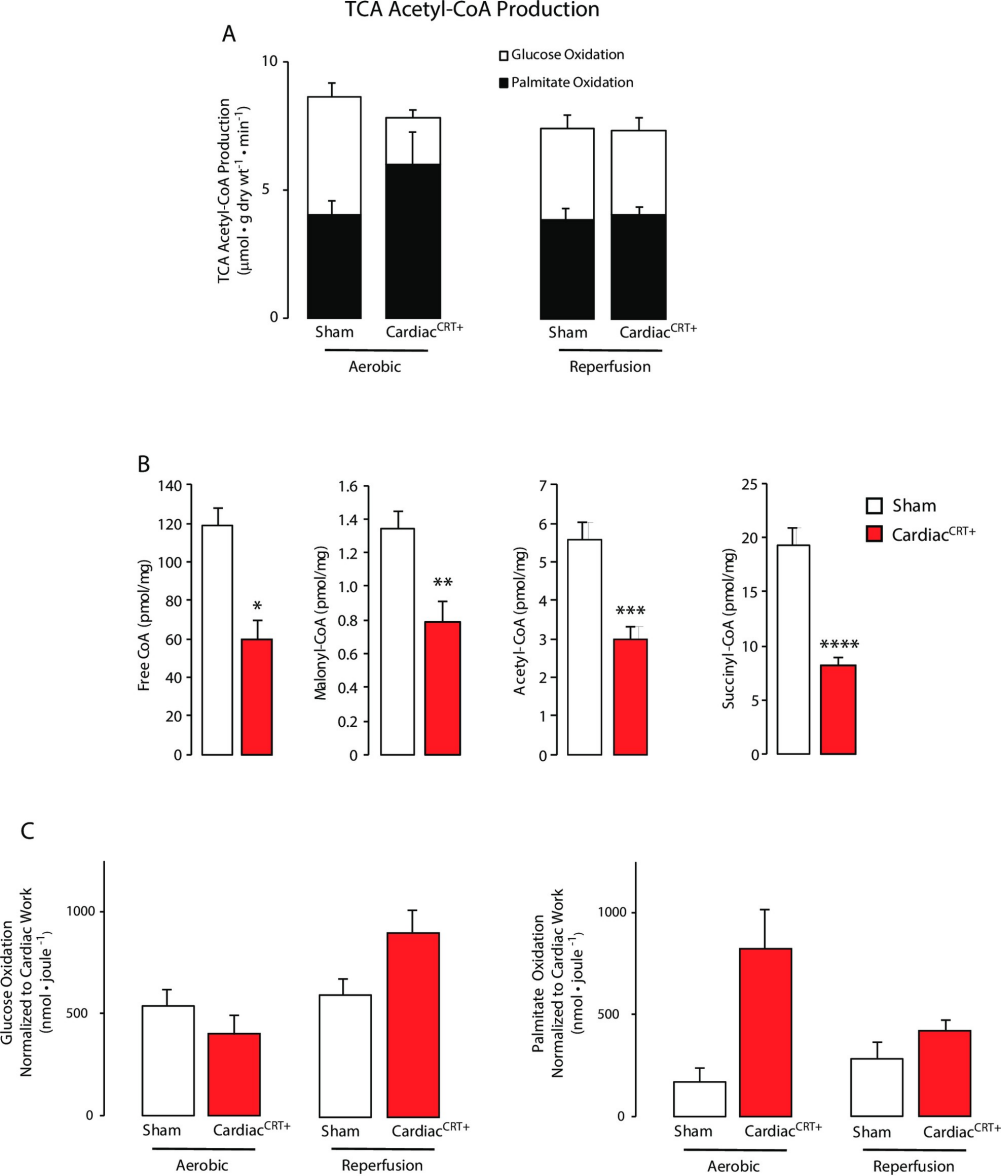
Cardiac glucose and palmitate oxidation. **A.** Assessment of acetyl-CoA production **B.** HPLC analysis of fatty acid CoA isoforms including free CoA, malonyl-CoA, acetyl-CoA and succinyl-CoA in sham control and Cardiac^CRT+^ heart samples frozen at the end of reperfusion. HPLC analysis was performed in triplicate. *p value = 0.015, **p value = 0.0255, ***p value = 0.379, ****p value = 0.0069. **C.** Measurement of radiolabeled glucose and palmitate during perfusion and reperfusion. *p value = 0.016.

Whole body calorimetry showed that Cardiac^CRT+^ mice were indolent (Fig 1A and 1B) which may be related to poor heart function. Thus, we subjected Cardiac^CRT+^ and sham control hearts to *ex vivo* aerobic perfusion and reperfusion to measure rates of glucose oxidation (Fig 2A), fatty acid oxidation (Fig 2B) and glycolysis (Fig 2C). During aerobic perfusion, the rate of palmitate oxidation in Cardiac^CRT+^ hearts were increased (Fig 2B), but the rate of glucose oxidation rate was decreased (Fig 2A). Furthermore, the total amount of ATP produced from glucose oxidation in Cardiac^CRT+^ hearts was lower than sham control hearts (Fig 2D). Consequently, cardiac efficiency of hearts with increased abundance of calreticulin selectively in cardiomyocytes was decreased by 40% compared with sham control hearts (Cardiac^CRT+^: 0.239 vs. sham control: 0.397 joules*μmol^-1^, respectively) (Fig 2E). Next, the hearts were aerobically reperfused (40 min) following no-flow ischemia (20 min). Acetyl-CoA production was increased in Cardiac^CRT+^ hearts with rate of glycolysis, and rates of palmitate and glucose oxidation exhibited similar patterns during aerobic perfusion (Fig 3A). Thus, to determine if Cardiac^CRT+^ hearts were dependent on fatty acid oxidation for energy production, coenzyme A was monitored. All isoforms of coenzyme A were reduced in Cardiac^CRT+^ hearts after ischemia (Fig 3B), indicative of reduced fatty acid oxidation. Utilization of radiolabeled glucose and palmitate by sham control and Cardiac^CRT+^ hearts (Fig 3C) indicated that sham control hearts switched to fatty acid oxidation whereas Cardiac^CRT+^ hearts continued and enhanced glucose oxidation (Fig 3C) during aerobic reperfusion. Therefore, overexpression of calreticulin in cardiomyocytes apparently improved functional recovery and cardio-protection of the heart.

### Enhanced cardiac recovery of Cardiac^CRT+^ hearts after ischemia

**Fig 4:**
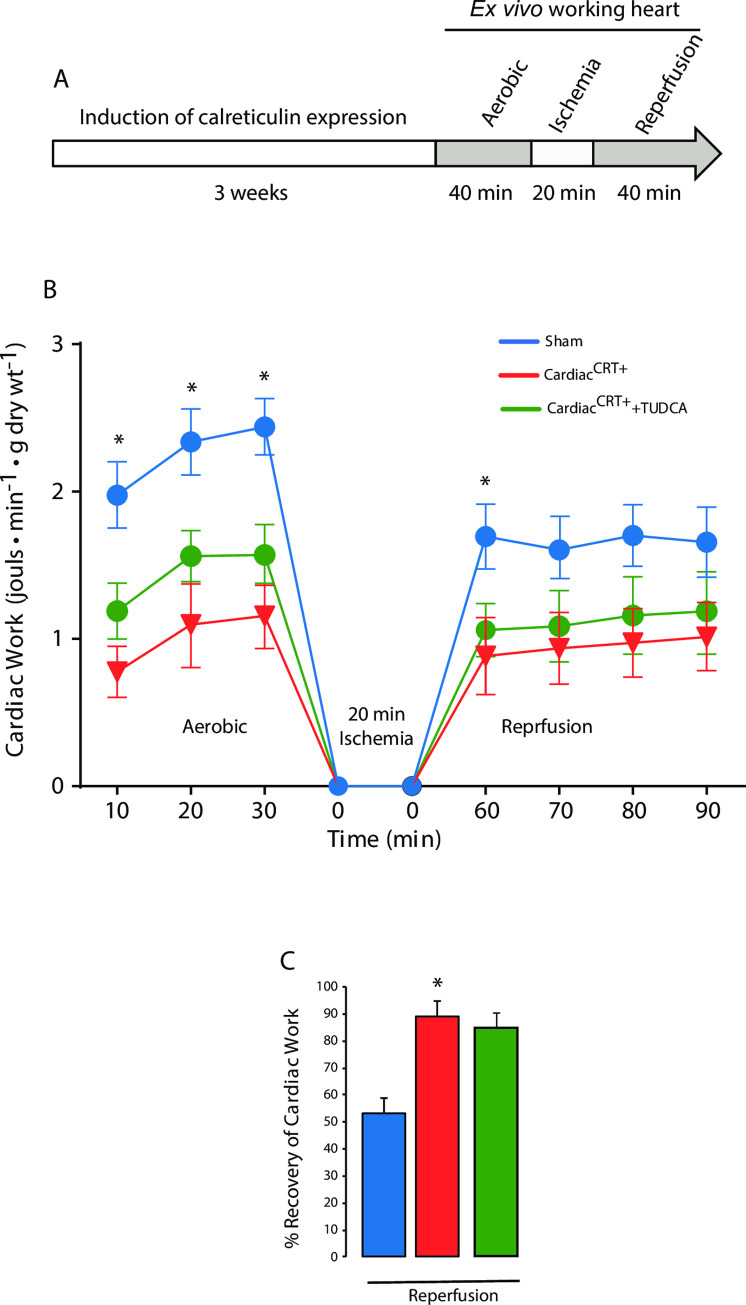
*Ex vivo* cardiac function in Cardiac^CRT+^ hearts. **A.** Schematic outline of the experimental procedure to analyze cardiac function and energy metabolism of Cardiac^CRT+^ and sham control mouse hearts. *Ex vivo* analysis of the working heart with 30 min aerobic perfusion, 20 min ischemia, and 40 min aerobic reperfusion using the Langendorff perfusion system. **B.** Cardiac work in aerobically reperfused hearts from Cardiac^CRT+^ and sham control mice was measured at 10 min intervals. Cardiac work was calculated by (PSP-11.5)*CO*133.322*0.000001*60s/60s/g dry wt. PSP = peak systolic pressure (mm Hg); 11.5 = the preload pressure (mm Hg) on mouse rig; CO = cardiac output (ml/min); 133.322 = 1 mmHg is 133.322 Pa = 133.322 kg/m•s2; 0.000001 = conversion factor (ml/min to 1•10–6 m^3^); 60 s convert to min to seconds; g dry wt = gram dry weight of the heart atria and ventricle. **C.** Cardiac work normalized to perfused heart during reperfusion. Cardiac work, % recovery in aerobically reperfused hearts from Cardiac^CRT+^ and sham control hearts. The % recovery is given by the ratio of perfused and reperfused cardiac work (COxPSP). Values are mean ± SEM, n = 20 (Cardiac^CRT+^), n = 25 (sham control), n = 6 (Cardiac^CRT+^+TUDCA). *p value<0.0001.

We used an *ex vivo* perfused working heart technique to assess cardiac performance of Cardiac^CRT+^ hearts (Fig 4A). In agreement with previous observations in the whole animal model [16], Cardiac^CRT+^ hearts had decreased peak systolic pressure (PSP), cardiac output, and aortic outflow, consistent with impaired left ventricular systolic function (Table 3). Furthermore, these hearts displayed lower cardiac power during aerobic perfusion for 30 min (Fig 4B), which was improved during aerobic reperfusion (Fig 4B). Mechanical function of Cardiac^CRT+^ hearts was better after ischemic/reperfusion injury as compared to sham control hearts (Fig 4B and 4C). Moreover, Cardiac^CRT+^ hearts had higher recovery rates after global ischemia than sham control hearts (Cardiac^CRT+^: 89.2±5.45% vs. sham control: 53.2±5.84%, Fig 4B and 4C). TUDCA treatment had no effect on Cardiac^CRT+^ cardiac function during ischemia or reperfusion indicating that the effect of this drug previously observed *in vivo* [17] is not directed towards the mechanical function of the heart (Fig 4B and 4C).

**Table 3:** Mechanical function of sham control and Cardiac^CRT+^ hearts.

Cardiac function (perfusion)	Sham control n = 31	Cardiac^CRT+^ n = 30	TUDCA n = 5	Cardiac^CRT+^+TUDCA n = 7
Heart rate (bpm)	314±73	269±6.4	318±17.9	325±26
Peak systolic pressure (mm Hg)	72±0.9	63.9±1.0	66.2±3.2	63.2±1.5
Developed pressure (mm Hg)	30±1	21.7±1.4	20.9±2.6	14.7±1.5
HR x PSP (10^−3^)	22±0. 5	18.9±0.45	20.9±0.78	20.4±1.47
HR x PSP (10^−3^)	9.4±0.3	6.36±0.4	6.49±0.6	4.65±0.4
Cardiac output (ml/min)	11.4±0.31	8.01±0.59	9.45±1.48	7.18±1.03
Aortic outflow (ml/min)	8.5±0.29	4.9±0.6	6.57±0.7	3.24±0.4
Coronary flow (ml/min)	2.9±0.28	3.14±0.32	2.88±1.02	3.18±0.81
Cardiac work (ml*mmHg/min)	8.2±0.24	5.18±0.42	6.44±1.23	4.49±0.61
Cardiac work (%)	100	100	100	100
Cardiac power (ml*mmHg/min)	2.83±0.13	1.96±0.2	2.01±0.4	1.44±0.18
Cardiac power (%)	100	100	100	100
**Cardiac function (reperfusion)**	n = 31	n = 30	n = 5	n = 7
Heart rate (bpm)	233±11.6	254±7	232±8.2	309±18
Peak systolic pressure (mm Hg)	62±2.4	64.7±1.0	55.9±6.3	63.0±0.9
Developed pressure (mm Hg)	22.0±1.9	21.9±1.2	14.2±1.5	13.7±0.6
HR x PSP (10^−3^)	15±1	16.6±0.62	16.6±0.62	19.5±1.19
HR x PSP (10^−3^)	5.6±0.6	5.62±0.4	3.98±0.6	4.3±0.4
Cardiac output (ml/min)	6.4±0.64	6.68±0.54	6.21±1.09	6.42±0.95
Aortic outflow (ml/min)	3.4±0.5	3.95±0.5	4.17±0.1	3.02±0.2
Coronary flow (ml/min)	2.7±0.27	2.72±0.26	3.08±0.73	2.58±0.51
Cardiac work (ml*mm Hg/min)	4.3±0.48	4.2±0. 4	4.68±0.7	4.03±0.6
Cardiac work (%)	53.2±5.84	89.2±5.45	66.4±3.16	84.1±4.62
Cardiac power (ml*mm Hg/min)	1.5±1	1.68±0.19	1.59±0.363	1.28±0.19
Cardiac power (%)	52.5±5.929	89.66±5.57	65.8±2.91	84.5±4.65

Values are mean ± SE

### Cardiac^CRT+^ cardiomyocytes exhibit improved cardiomyocyte function *in vitro*

**Fig 5:**
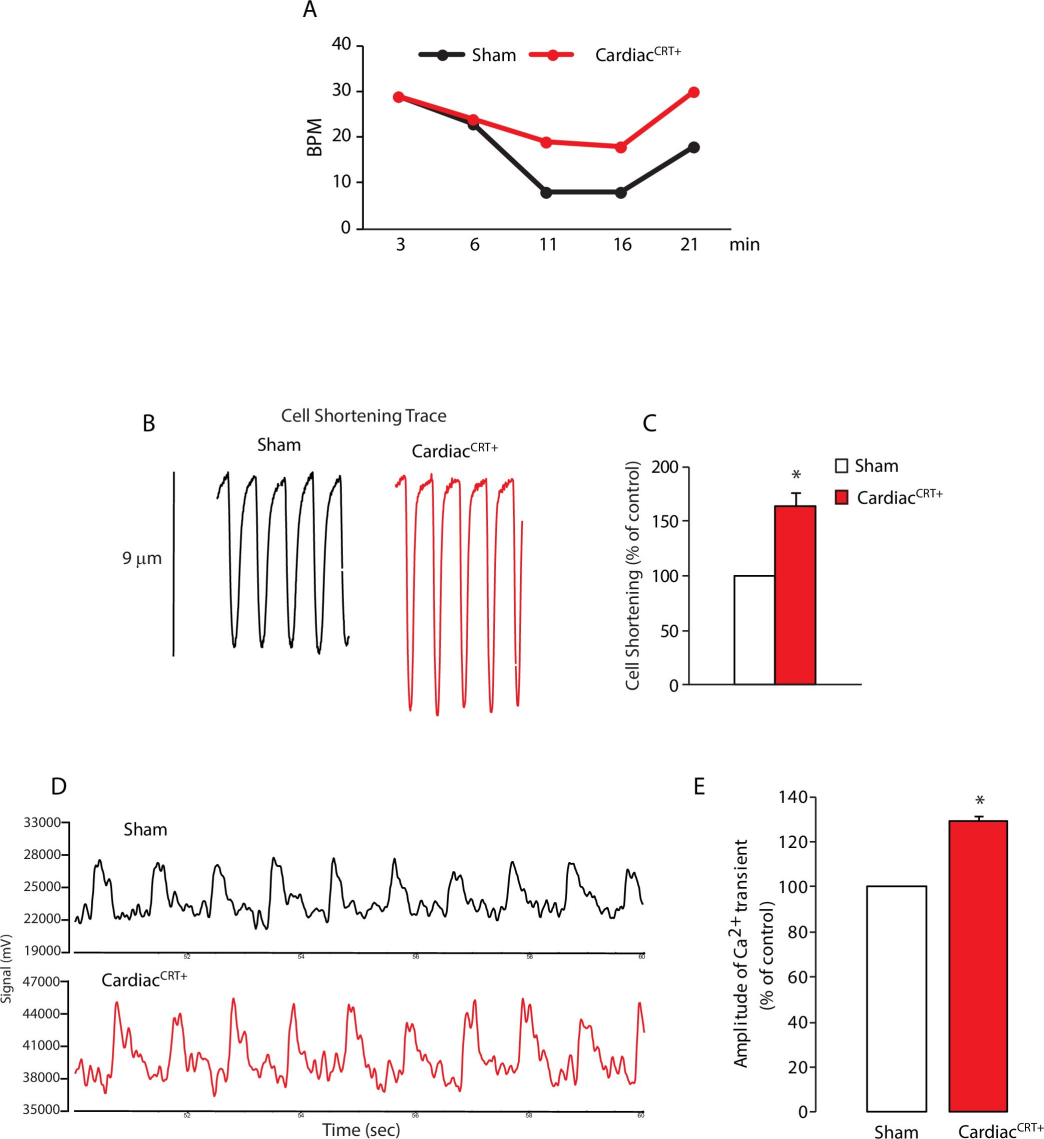
Functional analysis and Ca^2+^ transients in Cardiac^CRT+^ cardiomyocytes. **A.** Beats per minute (BPM) was monitored for the *ex vivo* hearts over time. **B.** Cell shortening traces were monitored for sham and Cardiac^CRT+^ cardiomyocytes. **C.** Cell shortening plotted as % of sham controls. Sham controls (n = 12), Cardiac^CRT+^ (n = 12); values are mean ± SEM. *p value<0.0001. **D.** Representative Ca^2+^ transients were monitored using Calcium Green-1/AM. Sham control (n = 12), Cardiac^CRT+^ (n = 12). **E.** Amplitude of Ca^2+^ transients are expressed as % of sham. Sham control (n = 12), Cardiac^CRT+^ (n = 12); values are mean ± SEM.

We isolated cardiomyocytes from sham control hearts and Cardiac^CRT+^ failing hearts and evaluated their performance by comparing their contractile activity and Ca^2+^ transients. Notably, Cardiac^CRT+^ cardiomyocytes exhibited higher beat frequency (by 50%) (Fig 5A) compared to sham control cardiomyocytes. Furthermore, the Cardiac^CRT+^ cardiomyocytes showed longer contraction distance (Fig 5B and 5C; S1 Video), indicative of robust contractility as compared to sham control cardiomyocytes. Analysis of Ca^2+^ transients revealed that Cardiac^CRT+^ cardiomyocytes had increased Ca^2+^ transient amplitudes (by 20%) (Fig 5D and 5E). These results demonstrate that cardiomyocytes isolated from Cardiac^CRT+^ hearts unexpectedly had increased contractility, beat frequency and Ca^2+^ transients.

### Activation of the IRE1α signaling in stretch-induced cardiac fibroblasts is prevented by TUDCA

**Fig 6:**
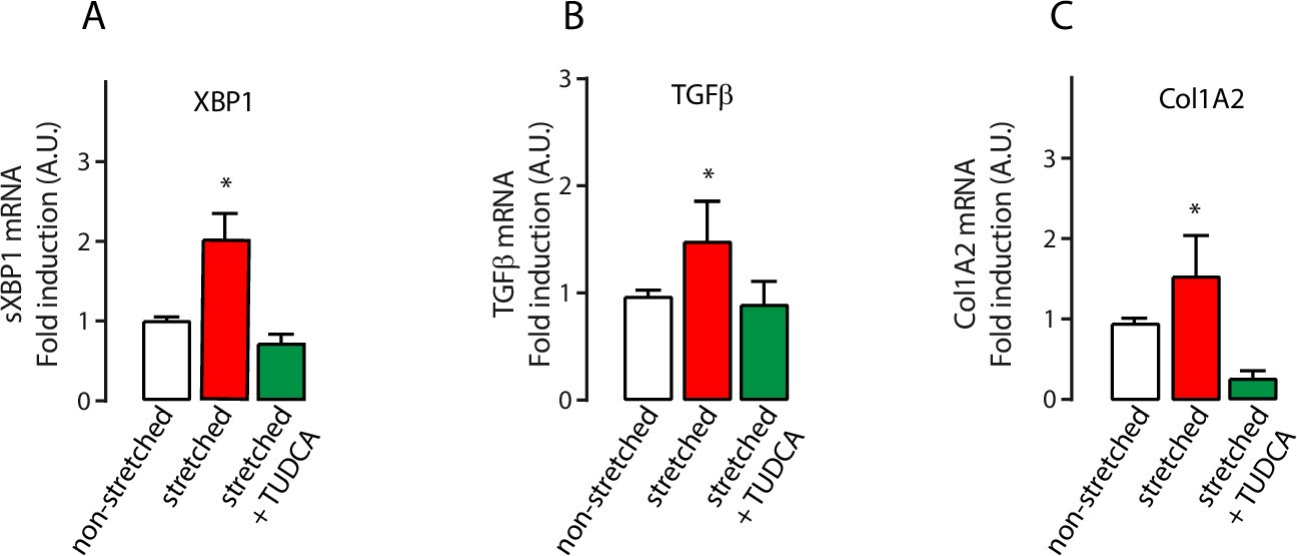
XBP1 splicing and fibrosis markers in mechanically stretched cardiac fibroblasts. RNA abundance of selected markers in non-stretched and stretched fibroblasts in the absence or presence of TUDCA. **A,** spliced XBP1 (*sXBP1*) mRNA. *p value = 0.029 (n = 5); **B.** TGFβ1 mRNA *p value = 0.034 (n = 5); **C.** collagen 1A2 mRNA. *p value = 0.031 (n = 5).

While overexpression of calreticulin improved cardiomyocyte function in Cardiac^CRT+^ hearts, these hearts paradoxically feature severe cardiac pathology characterized by fibrosis, irregular ECG and heart failure, (S1 Fig) [16, 17]. Notably, cardiac fibrogenesis was inhibited by TUDCA after selective induction of calreticulin overexpression in the murine cardiomyocytes *in vivo* [17]. The heart is comprised of multiple cell types that work together to carry out organ function [1]. Fibrogenesis is associated with activation of cardiac fibroblasts in response to overwork and is initially an adaptive response to mechanical stress [28]. In Cardiac^CRT+^ hearts, the enhanced function of calreticulin-overexpressing cardiomyocytes imposes substantial mechanical stress on other cardiac cells that are not overexpressing calreticulin, including cardiac fibroblasts. To evaluate this situation *ex vivo*, we isolated cardiac fibroblasts from sham control hearts and subjected these cells to mechanical stretch. As shown in the Fig 6, stretching of cardiac fibroblasts caused the activation of IRE1α signaling as illustrated by splicing of XBP1 mRNA as well as the induction of TGF1β and Col1A2 gene expression, all of which were strikingly prevented by TUDCA.

## Discussion

Calreticulin is the major ER Ca^2+^ storage protein [29, 30]. The protein is highly expressed in embryonic heart and despite this, the expression of calreticulin is sharply downregulated in adult cardiomyocytes [9] which rely on Ca^2+^ to carry out its mechanical function in the heart [1]. In humans, studies have noted increased expression of calreticulin in the failing and hypertrophied hearts [11–15]. We previously demonstrated experimentally in mice that overexpression of calreticulin in Cardiac^CRT+^ cardiomyocytes led to development of severe cardiac pathology characterized by cardiac hypertrophy, cardiomyopathy, cardiac fibrosis and heart failure [16, 17]. Echocardiography analysis of Cardiac^CRT+^ hearts revealed impaired left ventricular systolic and diastolic function and impaired mitral valve function [16]. Oral administration of TUDCA, a proteostasis promoter [19], remarkably inhibited cardiac fibrogenesis in the failing hearts of Cardiac^CRT+^ mice and delayed heart failure [17], yet paradoxically the treatment did not ameliorate other cardiac clinical features associated with the failing Cardiac^CRT+^ heart. Regardless, the inhibition of fibrosis by TUDCA was highly beneficial since this delayed heart failure allowed cardiac function in treated Cardiac^CRT+^ mice to continue enabling these mice to maintain their physical capacity as compared to untreated Cardiac^CRT+^ mice.

Our objective was to gain insight into the nature of the cause of heart failure in Cardiac^CRT+^ mice with an increased level of calreticulin in adult cardiomyocytes. We carried out a series of analyses spanning the whole-body and cardiac cell fucntion to visualize the consequences of the ectopic calreticulin expression in the adult heart. Cardiac^CRT+^ mice became lethargic, as would be expected of mice with deteriorating heart function. Surprisingly, however, when tested *ex vivo*, the hearts from Cardiac^CRT+^ mice showed improved functional recovery and cardio-protection even after global ischemia as compared to hearts from sham control mice. Moreover, Cardiac^CRT+^ cardiomyocytes that were isolated from the failing hearts exhibited robust contractile ability and functional capacity that well exceeded that of sham control cardiomyocytes. Thus, it is apparent that while overexpression of calreticulin substantially improved the functional quality of Cardiac^CRT+^ cardiomyocytes, the benefits do not translate to increased overall function and efficiency of the whole organ. This may be related to the effects of calreticulin overexpression on cardiac energy metabolism. Hearts from Cardiac^CRT+^ mice were more reliant on fatty acid oxidation as a source of energy (Fig 2B and 2D), which paralleled the increase in whole body reliance on fatty acid oxidation (Fig 1E). This increased reliance on fatty acid oxidation was accompanied by a decrease in cardiac glucose oxidation rates (Fig 2A). This switch in oxidative metabolism may have contributed to the impaired contractile function seen in the Cardiac^CRT+^ mice, as an increase in fatty acid oxidation and a decrease in glucose oxidation is associated with impaired contractile function [7]. It is not clear how overexpression of calreticulin affected fatty acid oxidation and glucose oxidation, although it is clear that calcium is important in controlling both processes [7, 31].

Ca^2+^ plays a central role in cardiac physiology. Ca^2+^-handling proteins associated with ER/SR membrane are critical for muscle contraction and relaxation [32]. Just like other cells, cardiomyocytes have a network of perinuclear ER but they also possess the SR which represents a specialized form of the ER responsible for regulation of excitation-contraction coupling [1, 8, 33, 34]. The SR plays a key role in maintaining cardiac excitation–contraction coupling and mechanical function of cardiomyocytes [8] while the ER is the driver of intracellular Ca2+-dependent signal transduction and cellular Ca2+ homeostasis [8]. Even modest changes in the SR or ER Ca2+ signaling and handling alter cardiomyocyte function in time [35]. Increased abundance of calreticulin leads to increased cellular Ca^2+^ capacity, increased Ca^2+^ fluxes across ER and delayed Ca^2+^ store-operated Ca^2+^ entry [18]. Accordingly, we speculate that the enhanced mechanical performance of cardiomyocytes is attributable to increased Ca^2+^ capacity of ER of the Cardiac^CRT+^ cardiomyocytes making them less dependent on external Ca^2+^ (Ca^2+^-induced Ca^2+^ release) that is predominantly controlled by the release of Ca^2+^ from the SR. It is interesting to note that the increased expression of calreticulin in Cardiac^CRT+^ cardiomyocytes is accompanied by a corresponding decrease in the abundance of calsequestrin [16], the major Ca^2+^ handling protein of the junctional SR. However, hearts of mice with reduced SR Ca^2+^ capacity due to decreased abundance or lack of calsequestrin are highly sensitive to catecholamine-induced cardiac arrhythmias [36, 37]. Our findings suggest that there are two distinct and independent Ca^2+^ pools in cardiomyocytes that differ in their mechanisms of Ca^2+^ handling. The SR Ca^2+^ pool is mainly involved in driving cardiomyocyte mechanical function which when impaired leads to cardiac arrhythmias, whereas the ER Ca^2+^ pool is critical for maintenance of cellular homeostasis. We propose that the increased ER Ca^2+^ capacity and pool size of Cardiac^CRT+^ cardiomyocytes induced by calreticulin overexpression enhanced the ability of these cells to cope with cellular stress.

We showed previously that ectopic expression of calreticulin in Cardiac^CRT+^ hearts results in the activation of the IRE1α branch of the UPR [16, 17], and this is associated with cardiac fibrosis and heart failure. It is also interesting to note the higher prevalence of cardiac fibrosis among endurance athletes where enhanced cardiac performance is required [38–40]. UPR activation in cardiac fibroblasts results in release of pro-inflammatory cytokines, increased deposition of collagen and other extracellular matrix proteins [41] and contributes to a process of phenotypic remodelling [17, 42, 43]. The increased mechanical load in Cardiac^CRT+^ hearts caused by the enhanced contractility of Cardiac^CRT+^ cardiomyocytes imposes mechanical stress on neighboring cardiac fibroblasts, and we demonstrated here experimentally that the mechanical stress experienced by cardiac fibroblasts simulated *ex vivo* by stretching of the cultured fibroblasts caused activation of IRE1α signaling associated with activation of cardiac fibrosis. Importantly, this was prevented in cultured fibroblasts stretched in the presence of TUDCA. These findings provide support for the notion that coping strategy outcomes of the different cardiac cell types contribute to the complexity of cardiac disorders [1] as TUDCA treatment is effective in managing ER stress in fibroblasts to prevent fibrosis in Cardiac^CRT+^ hearts [17] but offered little therapeutic benefit to the other observed cardiac pathologies. Thus, a possible mechanism underlying the induction of heart pathology in Cardiac^CRT+^ mice, despite improved Cardiac^CRT+^ cardiomyocyte performance, is the unequal sensing of cellular stress and/or activation of stress coping strategies among the different cell types that make up the Cardiac^CRT+^ heart, leading to failure in the coordination of various cellular function at the organ level.

In summary, this study examined the consequences of calreticulin-overexpressing cardiomyocytes on heart health. We found that increasing the abundance of calreticulin in cardiomyocytes promoted cardiomyocyte function and enabled hearts with calreticulin-overexpressing cardiomyocytes to tolerate global ischemia as measured *ex vivo*. However, *in vivo*, hearts with calreticulin-overexpressing cardiomyocytes experience progressive deterioration of function culminating in heart failure. Increased mechanical load of hearts with calreticulin overexpressing hearts due to increased contractility of cardiomyocytes imposes mechanical stress on neighboring cardiac fibroblasts leading to cardiac remodelling. We surmise that the selective enhancement of cardiomyocyte performance is unmatched by other cardiac cell types with unaltered calreticulin abundance thereby causing stress and consequently resulting in pathological outcome.

## Supporting information

S1 Fig Histology, Echocardiography and ECG analyses of sham control, Cardiac^CRT+^, and Cardiac^CRT+^+TUDCA hearts. (PDF)

S1 Video Beating cardiomyocytes. (AVI)

S1 Dataset (ZIP)
