# Supplementary material for: Selective enhancement of cardiomyocyte efficiency results in a pernicious heart condition
Source: PLoS One. 2020 Aug 13;15(8):e0236457. doi: 10.1371/journal.pone.0236457 (PMC7425937; doi:10.1371/journal.pone.0236457)
Supplement: S1 Fig — (PDF) [file pone.0236457.s001.pdf]

A

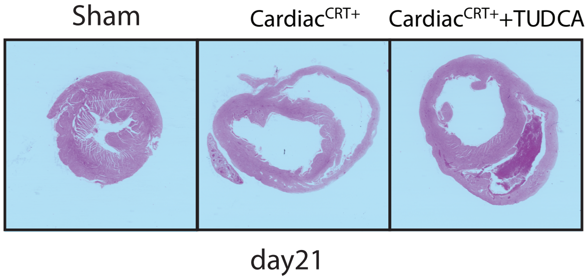

B

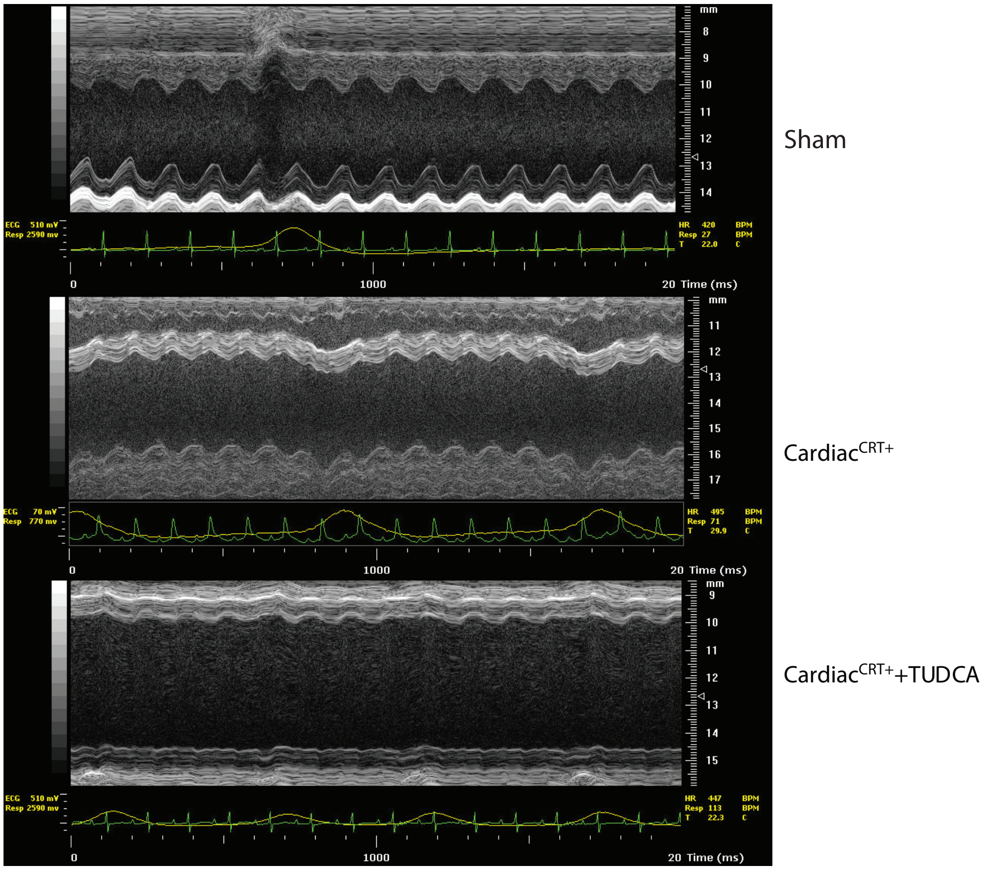

C

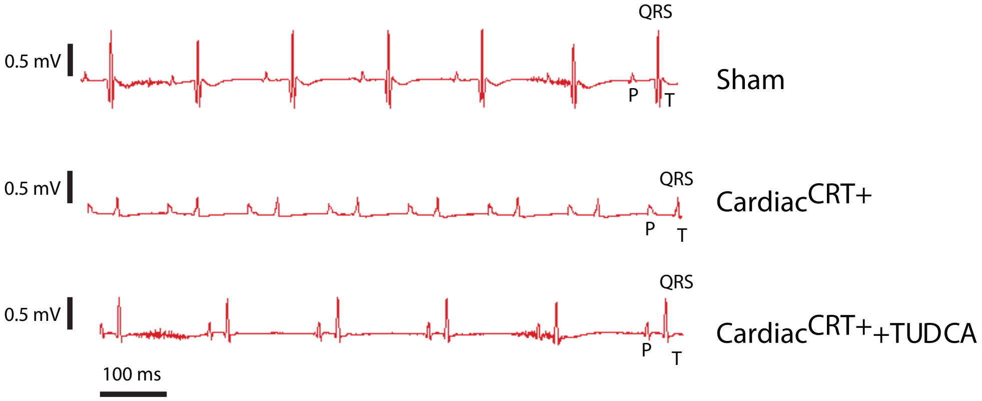

Supplemental Information Figure S1. Histology, Echocardiography and ECG analyses of sham control, Cardiac^{CRT+}, and Cardiac^{CRT+}+TUDCA hearts.

A. Heart histology was performed on Day 21 of Tamoxifen-treated sham control, Cardiac^{CRT+}, and Cardiac^{CRT+}+TUDCA-treated hearts.

B. Echocardiogram was performed on sham control, Cardiac^{CRT+}, and Cardiac^{CRT+}+TUDCA mice at Day 21 of Tamoxifen treatment.

C. ECG analysis of sham control, Cardiac^{CRT+}, and Cardiac^{CRT+}+TUDCA treated hearts. Sham control (n=6), Cardiac^{CRT+} (n=6), Cardiac^{CRT+}+TUDCA (n=6), numbers are mean ± SEM.
